# Synaptic E3 Ligase SCRAPPER in Contextual Fear Conditioning: Extensive Behavioral Phenotyping of *Scrapper* Heterozygote and Overexpressing Mutant Mice

**DOI:** 10.1371/journal.pone.0017317

**Published:** 2011-02-24

**Authors:** Ikuko Yao, Keizo Takao, Tsuyoshi Miyakawa, Seiji Ito, Mitsutoshi Setou

**Affiliations:** 1 Department of Medical Chemistry, Kansai Medical University, Moriguchi, Osaka, Japan; 2 Mitsubishi Kagaku Institute of Life Sciences, Machida, Tokyo, Japan; 3 Genetic Engineering and Functional Genomics Group, Frontier Technology Center, Graduate School of Medicine Kyoto University, Kyoto, Japan; 4 Section of Behavior Analysis, Center for Genetic Analysis of Behavior, National Institute for Physiological Sciences, Okazaki, Aichi, Japan; 5 Division of Systems Medical Science, Institute for Comprehensive Medical Science, Fujita Health University, Toyoake, Aichi, Japan; 6 Department of Molecular Anatomy, Hamamatsu University School of Medicine, Hamamatsu, Shizuoka, Japan; University of Queensland, Australia

## Abstract

SCRAPPER, an F-box protein coded by FBXL20, is a subunit of SCF type E3 ubiquitin ligase. SCRAPPER localizes synapses and directly binds to Rab3-interacting molecule 1 (RIM1), an essential factor for synaptic vesicle release, thus it regulates neural transmission via RIM1 degradation. A defect in SCRAPPER leads to neurotransmission abnormalities, which could subsequently result in neurodegenerative phenotypes. Because it is likely that the alteration of neural transmission in *Scrapper* mutant mice affect their systemic condition, we have analyzed the behavioral phenotypes of mice with decreased or increased the amount of SCRAPPER. We carried out a series of behavioral test batteries for *Scrapper* mutant mice. *Scrapper* transgenic mice overexpressing SCRAPPER in the hippocampus did not show any significant difference in every test argued in this manuscript by comparison with wild-type mice. On the other hand, heterozygotes of *Scrapper* knockout [SCR (+/−)] mice showed significant difference in the contextual but not cued fear conditioning test. In addition, SCR (+/−) mice altered in some tests reflecting anxiety, which implies the loss of functions of SCRAPPER in the hippocampus. The behavioral phenotypes of *Scrapper* mutant mice suggest that molecular degradation conferred by SCRAPPER play important roles in hippocampal-dependent fear memory formation.

## Introduction

SCRAPPER is a synapse-localized E3 ubiquitin ligase that was identified by *in silico* screening by us [1]. Pre- and post-synaptic sites contain complexes of scaffolding proteins, neurotransmitter-releasing machinery, receptors, ion channels and signaling molecules [2], [3], [4], [5]. Synaptic proteins are regulated by various sophisticated processes including control of transcription [6], [7], translation [8] and translocation [9], [10], [11]. Recently, protein degradation has attracted attention as a mechanism to control the amount of synaptic proteins. Selective proteasomal degradation is conducted by addition of ubiquitin to the target proteins by the enzymes of the ubiquitin-proteasome system (UPS) followed by protease digestion in the 26S proteasome [12], [13], [14], [15]. In the ubiquitin-mediated degradation pathway, E3 ubiquitin ligases play an essential role in the regulation of diverse biological processes by promoting degradation [14]. SCRAPPER directly binds to and ubiquitinates the active zone protein Rab3-interacting molecule 1 (RIM1) both *in vitro* and *in vivo*. Analyses of *Scrapper* mutant mice demonstrated that SCRAPPER-dependent UPS contributes to the regulation of synaptic vesicle release probability via RIM1 [1]. The target molecule RIM1 is an important factor for synaptic vesicle release and synaptic plasticity [2], [16], [17]. Thus, SCRAPPER regulates proper synaptic transmission via RIM1 degradation [1]. Through increased frequency of miniature excitatory post synaptic currents (mEPSCs) and reduced paired pulse facilitation, both of which are caused by increased release probability, we found that *Scrapper* knockout [SCR-KO or SCR (−/−)] hippocampal neurons had increased levels of spontaneously released neurotransmitter. Upregulation of synaptic vesicle release was induced by the proteasome inhibitor as well as in SCR-KO neurons. Such perceptions suggest that local protein degradation could be one of the regulatory mechanisms of neural transmission [1], [18], [19], [20].

To further reveal the physiological significance of neural transmission regulated by SCRAPPER *in vivo*, we analyzed 2 types of *Scrapper* mutant mice, namely, SCR-KO and *Scrapper* transgenic (SCR-TG) mice. Because of abnormalities of the SCR-KO neurons, as well as the significance of the target RIM1 functions for neural function, we suspected that SCR-KO mice may have behavioral abnormalities, particularly on learning and memory tasks. In this study, we investigated behaviors of *Scrapper* mutant mice according to an established comprehensive behavioral test battery often used on genetically engineered mice [21], [22], [23], [24], [25]. We found that the reduced amount of SCRAPPER was linked to the interference of fear conditioning to context, which means that SCRAPPER has a crucial role in fear conditioning. Coincidentally, several aspects of anxiety-related behavior were depressed in *Scrapper* mutant mice.

## Results

### General outline of the SCR (+/−) and SCR-TG mouse phenotypes

To understand the physiological role of SCRAPPER in the adult brain, we generated *Scrapper* gene-deficient and *Scrapper* gene-overexpressing mice [1]. Because of the lethality of C57/BL6J backcrossed null mutant mice, we analyzed heterozygous *Scrapper* gene-deficient mice [SCR (+/−)] using behavioral test battery. Western blot analysis showed a reduction of about 50% in SCR (+/−) whole brain lysates as described in our earlier report [1]. SCR (+/−) mice were almost the same size as the wild-type (WT) mice and were fertile, and grossly healthy; however, some heterozygous and null KO mice died suddenly in the early postnatal period from unknown reasons [1]. The body weight and temperature of the SCR (+/−) mice used in this study were almost the same as those of WT mice (Table 1).

**Table 1:** Comparison between *Scrapper*-knockout [SCR (+/−)] - and wild-type (WT) mice or *Scrapper*-transgenic (SCR-TG) - and WT mice.

Tests	Measurements	Meanings	Phenotypes	
			SCR (+/−)	TG
General health and neurological screening	Body weight	General health	→	
	Body temperature		→	→
	Wire hanging time		→	→
	Grip strength		→	→
Light/dark transition test **(** **Fig. 2** **)**	Transitions	Anxiety	→	→
Open field test **(** **Fig. 4** **)**	Total distance	Exploratory activity, affectivity,	→	→
	Vertical activity	anxiety	→	→
	Center time		↑	→
	Stereotypic counts		→	→
Elevated plus maze test **(** **Fig. 3** **)**	Entries	Anxiety	→	→
Hot plate test	Latency time	Pain sensitivity	→	→
Social interaction (novel environment)	Total duration of contacts	Social behavior,		→
**(** **Fig. 5** **)**	Number of contacts	anxiety-like behavior	→	→
	Total duration of active contacts		→	→
	Mean duration/contact			→
	Distance traveled		→	→
Social interaction (home cage) **(** **Fig. 6** **)**	Contacts	Social behavior, locomotor	↑ (at night)	N.A.
Social interaction (Crawley ver.)	Contacts	Social behavior,	→	N.A.
	General activity	anxiety-like behavior		N.A.
Rota-rod test	Latency time	Motor coordination	→	→
Acoustic startle response	Sound level	Auditory capacity	(110 dB)	→
Prepulse inhibition (PPI) test	Sound level	Sensorimotor gating	→	N.A.
Porsolt forced swimming test	Immobility time	Behavioral despair	→	→
Fear conditioning test **(** **Fig. 1** **)**	Immediate freezing during conditioning phase	Fear memory	↓	→
	Contextual testing conducted after conditioning		↓	→
	Cued test with altered context		→	→
Tail suspension test	Immobility time	Behavioral despair	→	
Barnes Maze	Spent time	Spatial working memory	→	N.A.
Gait analysis	Locomotion	Locomotion activity	→	N.A.
Eight-arm Radial Maze	Entries	Working memory	N.A.	→

→, no significant difference; ↑, increased, ↓, decreased in SCR (+/−) and wild-type (WT) mice or *Scrapper*-transgenic (SCR-TG) and WT mice.

Skew arrows show the increasing or decreasing tendency judged from the p-values (0.05<p<0.1). N.A., not analyzed.

In accordance with the other report [1], SCR-TG mice, which overexpress exogenous SCRAPPER protein in the hippocampus, had almost the same size and longevity as WT mice, although the former had tendency to light body mass [Table 1, genotype effect, p = 0.0975, f(1,30)  = 2.926] and less immobility in the tail suspension test [Table 1, genotype effect, p = 0.0518, f(1,30)  = 4.101]. Western blot analysis of SCR-TG hippocampal lysates showed about 1.2-fold upregulation of the SCRAPPER protein compared to that in lysates of WT mice [1]. SCR-TG mice appeared healthy and normal grossly normal. Because these mice showed no significant differences in the results of all the tests, except for body weight (Table 1), hereafter, we mainly describe the results of SCR (+/−) mice. The overall results of the present study are shown in Table 1 and the results of all tests and the values of each genotype effect are listed in supplemental data as Table S1.

SCR (+/−) mice showed significant difference in the following parameters when compared with WT mice; decreased freezing rate at the conditioning stage, increased time spent in the centre of the open field, reduced mean duration/contact time in the social interaction test in a novel environment, and increased activity levels with social interaction in the home cage test during the dark phase (Table 1).

### Reduced freezing of SCR (+/−) mice during fear conditioning

**Figure 1:**
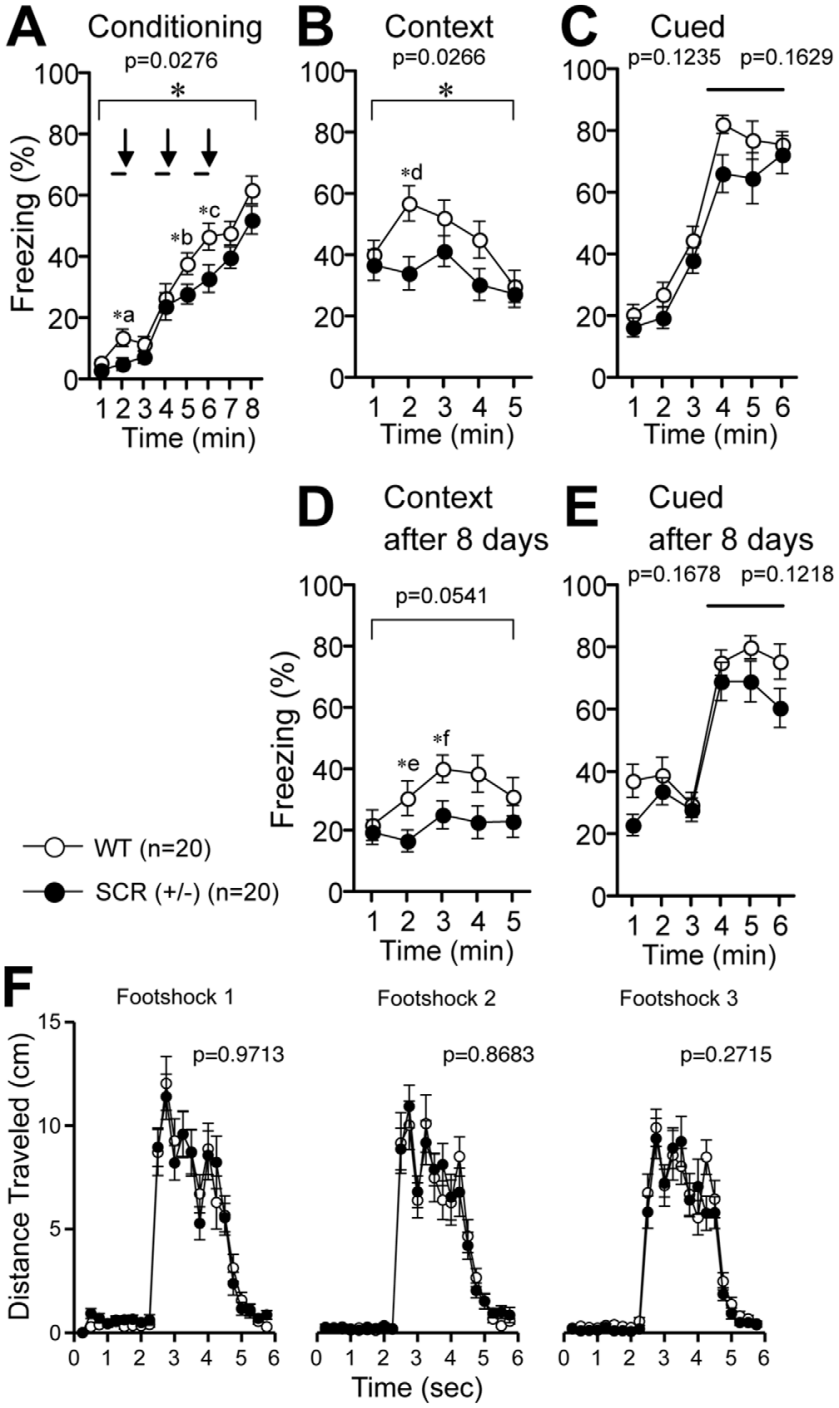
Contextual and cued fear conditioning in SCR (+/−) mice. (A) Percentage of time showing freezing during the training phase. A tone was presented for 30 s (bars) followed by a 2 s foot shock (arrows). (B) Reduced freezing was observed in the SCR (+/−) mice during the contextual test conducted at 24 h after conditioning. (C) Freezing during the tone-cued (bar) test with altered context in SCR (+/−) mice. (D, E) Retention of fear after 8 days. Percentage of time showing freezing during the contextual test in wild-type and SCR (+/−) mice (D) and freezing during the tone-cued (bar) test with altered context (E) at 8 days after conditioning. (F) Distance traveled soon after each foot shock at the training phase. (*) Significantly different in genotype effect, p<0.05. The p values indicate genotype effect in two-way repeated measures ANOVA (A–F). (*a–f) Post-hoc comparisons were performed using Fisher's PLSD multiple comparisons. (*a) p = 0.0187, (*b) p = 0.0454, (*c) p = 0.0367, (*d) p = 0.0064, (*e) p = 0.0440, (*f) p = 0.0244.

Among the test battery conducted in this study, the fear conditioning test showed the most significant difference between the 2 genotypes (Fig. 1). The cognitive functions of the SCR (+/−) mice and their WT littermates were analyzed in a contextual and cued fear conditioning test. During the conditioning period, SCR (+/−) mice showed lower levels of freezing after foot shocks [Fig. 1A, Conditioning, p = 0.0276, f(1,38)  = 5.246]. The effect of time was significant during conditioning in Fig. 1A for both genotypes [WT; p<0.0001, SCR (+/−); p<0.0001], while the Genotype × Time interaction was not [p = 0.4571, f(7,266) = 0.965]. In the post-hoc comparisons using Fisher's PLSD multiple comparisons for the results during the conditioning phase at 2, 5, and 6 min time point were significant [Fig. 1A, (*a) p = 0.0187, (*b) p = 0.0454, (*c) p = 0.0367]. In this regard, however, note that significance of (*a) is for 1 to 2 min block before the conditioning cue stimuli. In addition, there was no difference in the distance traveled soon after each foot shock at the training phase (Fig. 1F).

### Reduced freezing of SCR (+/−) mice in the contextual fear conditioning test

When the conditioned stimulus (context) was presented 24 h after conditioning (context testing), SCR (+/−) mice showed reduced levels of freezing [Fig. 1B, Contextual test after conditioning, p = 0.0266, f(1,38)  = 5.324]. We noted that at 8 days after conditioning, the difference between WT- and SCR (+/−) mice showed the similar tendency to the data of 24 h after conditioning [Fig. 1D, p = 0.0541, f(1,38)  = 3.951 at 8 days after]. In the post-hoc comparisons in the data of Fig. 1B, there was significance at the 2 min point [Fig. 1B, (*d) p = 0.0064, Fisher's PLSD test], and in Fig. 1D at the 2 and 3 min time point [Fig. 1D (*e) p = 0.0440, (*f) p = 0.0244, Fisher's PLSD test]. The effect of time was significant during contextual testing after 24 h (Fig. 1B) in WT but not in SCR (+/−) [WT; p = 0.0013, SCR (+/−); p = 0.1943], while the time interaction was not significant between 2 genotypes [p = 0.1527, f(4,152)  = 1.701]. Eight days after conditioning (Fig. 1D), the effect of time was also significant only for WT mice [WT; p = 0.0079, SCR (+/−); p = 0.4197], and the time interaction was not significant [p = 0.2799, f(4,152)  = 1.281.

When the conditioned stimulus (tone) was presented in an altered context both 24 h and 8 days after conditioning (cued test), SCR (+/−) mice did not show significant differences in the levels of freezing [24 h; Fig. 1C, Cued test with altered context, p = 0.1629, f(1,38)  = 2.025, 8 days after; Fig. 1E, p = 0.1218, f(1,38)  = 2.505]. Thus, the results of the fear conditioning test shown in Fig. 1 suggest that the contextual fear conditioning in SCR (+/−) mice was selectively impaired.

### Tendency to have reduced responses in the prepulse inhibition test

To know whether the reduced freezing in the fear conditioning test was affected by abnormalities in the sensory-motor impairment, we reviewed the results of the acoustic startle response, prepulse inhibition test, Porsolt forced swim test, and Barnes maze test. The acoustic startle response of the SCR (+/−) mice was not impaired significantly, although we noted that the response with 110 dB sound tends to be reduced [Table S1; 110 dB, p = 0.0728, f(1,38)  = 3.406; 120 dB, p = 0.1730, f(1,38)  = 1.929]. Responses in the prepulse inhibition test, which often reflects psychiatric disturbance, were not significant in the mutant mice (Table S1). We also noted that among the 4 stimuli in the prepulse inhibition test, there was reduced tendency but not significant under 1 condition [Table S1, 110 dB startle, prepulse sound level 78 dB, p = 0.0507, f(1,38)  = 4.071]. These results show that the SCR (+/−) mice could hear sounds and the reduced freezing rate in the SCR (+/−) mice was not derived from the hearing loss. In the Porsolt forced swim test which is a depression model [26], also known as the behavioral despair test, SCR (+/−) mice behaved similar to WT mice (Table S1). In the Barnes maze test with 24 h to 35 days retention was conducted to test the spatial working memory of SCR (+/−) mice; they showed normal behavior (Table S1).

### Time spent in the centre of the open field

**Figure 2:**
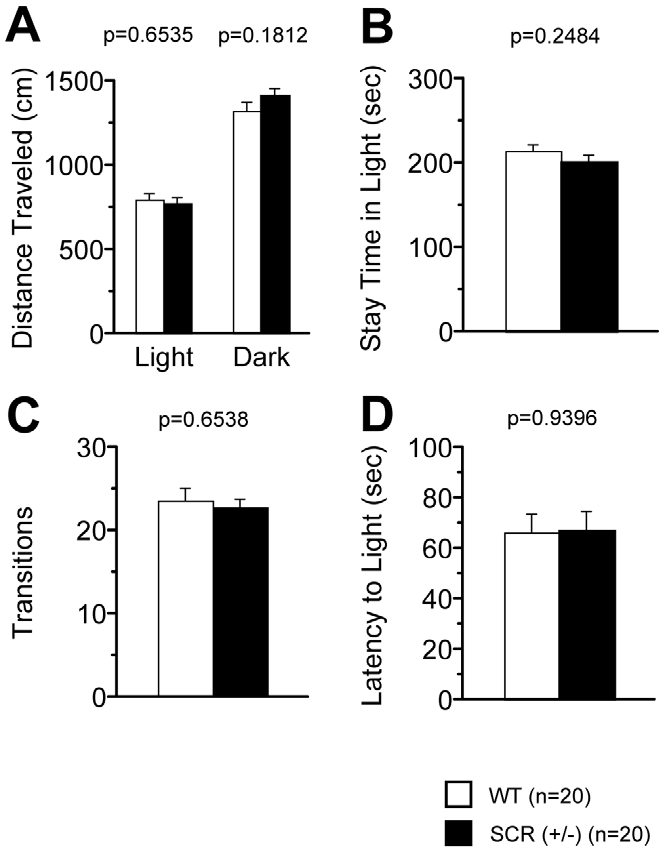
Normal behaviour of light/dark transition in SCR(+/−) mice. The distance traveled (A), the stay time in the light chamber (B), the number of transition times (C), and the latency of the light (D) were similar in SCR (+/−) compared with wild-type mice. The p values indicate genotype effect in two-way ANOVA (A–D).

**Figure 3:**
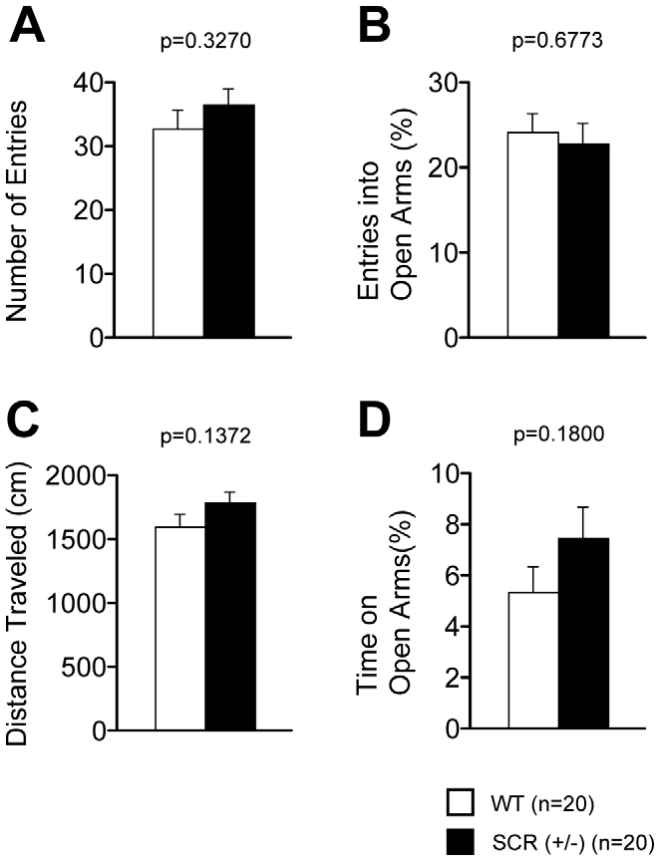
Normal behaviors of elevated plus maze in SCR(+/−) mice. The number of entries into the open arms (A), the rate of entries into open arms (B), the distance traveled during the elevated plus maze test (C), and the time on the open arms (D) were similar in SCR (+/−) compared with wild-type mice. The p values indicate genotype effect in two-way ANOVA (A–D).

**Figure 4:**
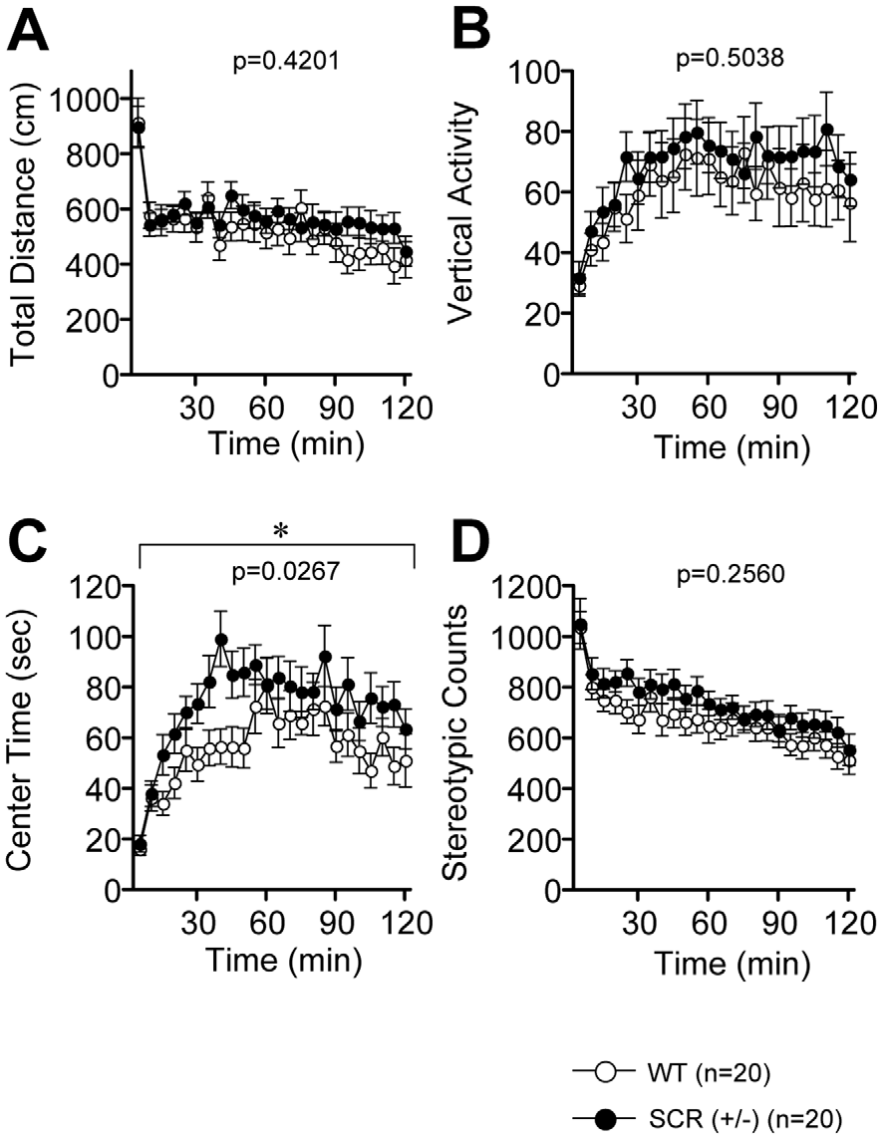
Time spent in the center in SCR(+/−) mice. Comparison between SCR (+/−) (mutants) and wild-type (controls) mice in the open field test with total distance (A), vertical activity (B), center time (C), and stereotypic counts (D). (*) Significantly different in genotype effect, p<0.05. The p values indicate genotype effect in two-way ANOVA (A–D).

Because we conducted a series of basic test battery and analyzed the behavior characteristics of the SCR (+/−) mice, we analyzed whether the results of the test battery were associated with fear conditioning. Among the basic neurophysiological characteristics of SCR (+/−) mice, there were no significant differences in neuromuscular strength (grip strength and wire hang test, Table 1), motor coordination (rotarod test, Table 1), pain sensitivity (hot plate test, Table 1), and gait pattern (gait analysis, Table 1). Among the assessment of anxiety, SCR (+/−) mice showed patterns similar to those of WT mice in the light/dark transition test (Fig. 2, p>0.1 for all) and in the elevated plus maze test (Fig. 3, p>0.1 for all). In the open field test, SCR (+/−) mice showed normal behavior with total distance (Fig. 4A, p>0.1), vertical activity (Fig. 4B, p>0.1), and stereotypic counts (Fig. 4D, p>0.1). There was a significant difference between the 2 genotypes in the time spent in the center of the open field apparatus [Fig. 4C, p = 0.0267, f(1,38)  = 5.314], which is usually thought to reflect reduced anxiety [27]. These results show that anxiety-related behaviors of SCR (+/−) mice were altered.

### Reduced mean duration/contact time in the social interaction test in a novel environment

**Figure 5:**
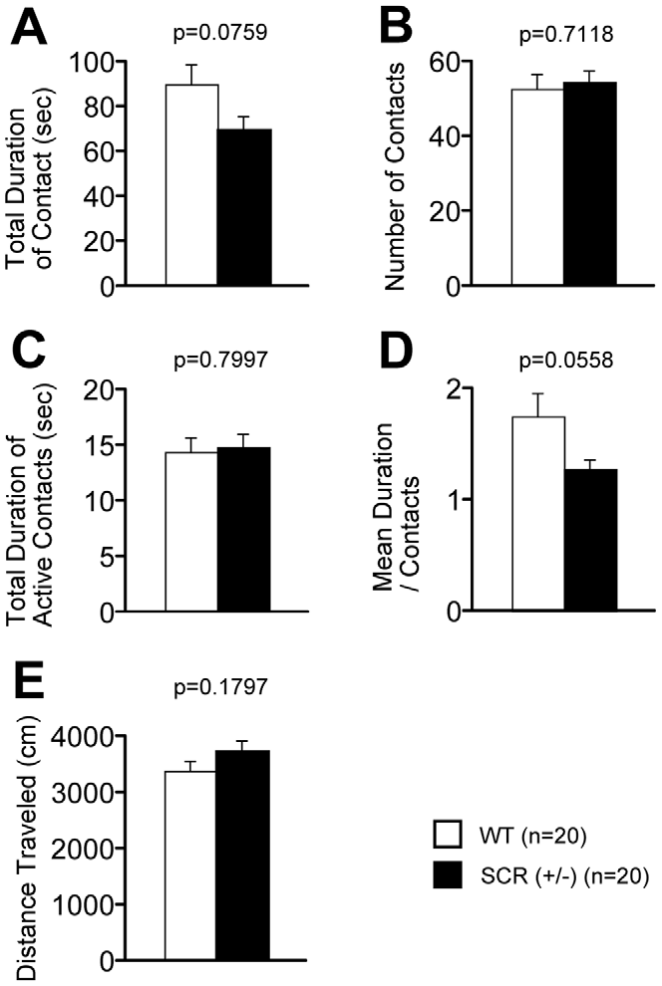
Decreased social behaviors in SCR (+/−) mice. (A–E) Social interaction test in a novel environment (one-chamber social interaction test): total duration of active contacts (A), number of contacts (B), total duration of active contacts (C), mean duration per each contact (D), and total distance traveled (E) were recorded. The p values indicate genotype effect in two-way ANOVA (A–E).

In the social interaction test, SCR (+/−) mice had reductions in both total duration and mean duration of contacts compared to their WT littermates but this failed to reach significance [Figs. 5A; Total duration of contacts, p = 0.0759, f(1,17)  = 3.572 and 5D; Mean duration/contact, p = 0.0558, f(1,17)  = 4.215]. The number of social contacts and the distance traveled did not differ significantly between the 2 genotypes [Figs. 5B, p = 0.7118, f(1,17)  = 0.141 and 5E, p = 0.1797, f(1,17)  = 1.958]. The duration of active contacts, which was counted if 2 mice contacted each other after either mouse traveled farther than 5 cm, was not different between the 2 genotypes [Fig. 5C, p = 0.7997, f(1,17)  = 0.066]. Because an increase in the number of social interactions is usually considered a measure of reduced anxiety [28], it is likely that the tendency of the decreased duration of contacts shown by the SCR (+/−) mice was primarily influenced by their downregulated anxiety.

Crawley's 3-chamber social approach test consists of a sociability test and a social novelty preference test, which is characterized by the preference of the mice based on the time spent around a wire cage containing a stranger mouse vs. an empty cage in the sociability test and a stranger mouse vs. a familiar mouse in the social novelty preference test [29]. In the sociability test, both SCR (+/−) mice and WT mice demonstrated normal sociability [Table S2; time spent around cage, with stranger vs. empty; WT: p = 0.315, SCR (+/−): p = 0.497, paired *t*-test]. SCR (+/−) mice showed the same behavior as WT mice with a preference for the chamber with the stranger [Table S2; time spent in chambers (stranger 1 side vs. empty cage side); WT mice: p = 0.8850; SCR (+/−) mice: p = 0.4314, paired *t*-test].

### Increased activity level at night with social interaction in the home cage test

**Figure 6:**
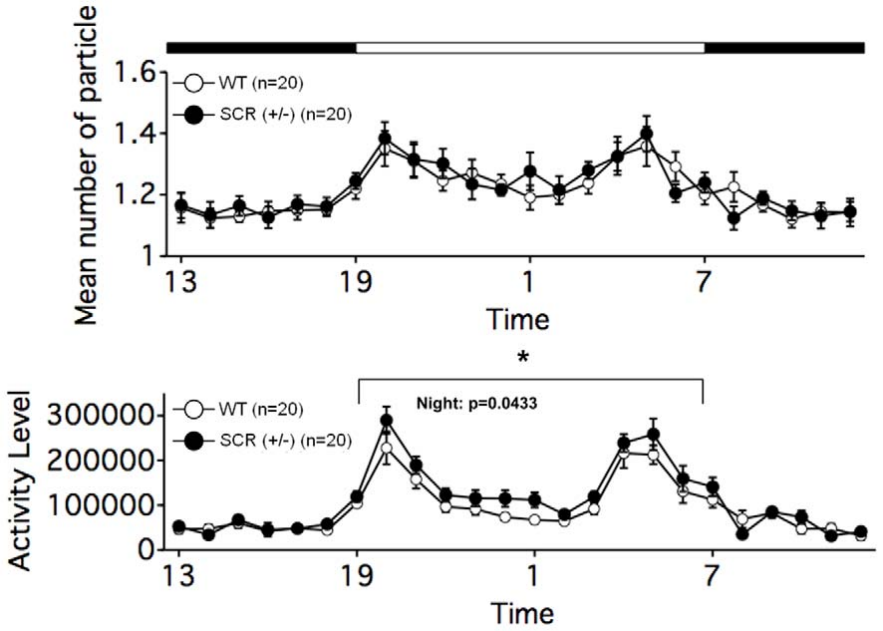
Increased social interaction activity in home cage in SCR (+/−) mice. Social interaction test in home cage: mean number of particles detected and activity level were recorded over 6 days. The graph shows the mean values of those recorded in the middle 3days. (*) The activity of mutant mice increased significantly throughout the dark period. The p values indicate genotype effect in two-way repeated measures ANOVA.

Lastly, the social interaction of SCR (+/−) mice in the home cage under familiar conditions was observed over a 6-day period. In the social interaction test in the home cage, time spent separated is usually increased when mice are active and decreased when mice are sleeping. Averaged 6-day period to 1-day data show that SCR (+/−) mice spent almost the same time separated from each other as WT mice [Fig. 6; genotype effect, p = 0.727, f(1,14)  = 0.127] and locomotor activity tended to be higher in SCR (+/−) mice [Fig. 6; genotype effect, p = 0.0643, f(1,14) = 4.035]. These phenotypes were observed only in the dark period [Fig.6, activity level, p = 0.0433, f(1,14)  = 4.935], although they were almost the same in the light period [Fig. 6; activity level, p = 0.6329, f(1,14)  = 0.238].

## Discussion

### SCR-TG and SCR (+/−) mice

In this study, we obtained the results from SCR-TG and SCR (+/−) mice. Although we successfully generated 100% backcrossed C57/BL6J knockout mice, they died within 1 day of birth. Therefore, we performed the behavioral test battery with heterozygote *Scrapper* gene knockout mice of the C57BL6J line [1]. It is likely that the null mutant mice would have a more significant difference in many tests if they had survived. Unfortunately, the mixed lines are not suitable for behavioral tests, as only a few 129sv/C57BL6J back line null mutant mice for *Scrapper* survived until adulthood [1], [30].

### Mechanisms of fear memory formation and involvement of SCRAPPER

The behavioral test battery revealed that SCR (+/−) mice were impaired in contextual fear conditioning. Pavlovian fear conditioning results in the formation of a strong association between a neural conditioned stimulus (CS; e.g., an audible sound) and an aversive unconditioned stimulus (US; e.g., a foot shock) that can trigger stereotypic fear responses [31], [32]. In fear conditioning, a fearful experience establishes a memory that can result in long-term behavioral changes. On the first day, mice are placed into a chamber and conditioned with a tone (CS) paired with a foot shock (US). On the second day, the mice are returned to the chamber and the incidences of freezing are examined in the absence of the tone and foot shock (context test). Alternatively, the mice can be placed in a novel chamber, where freezing behavior is noted after the presentation of the tone without the foot shock (cued test). In both tests, immobility or freezing behavior is scored [33]. By examining freezing behavior in different conditions, the dual mechanisms underlying conditional emotional memory can be examined [34], [35]. Freezing during the context test is attributed to hippocampal or temporal lobe processes [36]. Deficits in freezing during the context and cued tests are indicative of amygdala dysfunction [37], [38].

In both humans and experimental animals, emotional memory, as that learned fear, is critically dependent on the amygdala complex. [37], [39], [40]. In this study, SCR (+/−) mice showed significant differences in the contextual tests. SCR (+/−) mice had similar tendency to WT mice in the cued test both 24 h and 8 days after conditioning, suggesting the selective hippocampal involvement.

### Thalamic function within normal range in SCR (+/−) mice

The behavioral test battery revealed that SCR (+/−) mice had impaired ability of fear conditioning. From the PPI test, it became apparent that SCR (+/−) mice had almost normal sensation of hearing which is regulated by the thalamus. Thus, we evaluated that the reduced freezing was not affected by abnormalities in the sensory-motor impairment; i.e., SCR (+/−) mice could hear the tone in fear conditioning test, although they had the tendency to have downregulated response at 110 dB. The phenotype at 110 dB may contribute, or arise from the reduced anxiety in SCR (+/−) mice.

### Fear learning deficits during acquisition in SCR (+/−) mice

In the fear conditioning test, we found that the freezing rate was significantly reduced in SCR (+/−) mice during the conditioning phase. The results show the fear learning deficits during acquisition, that is, acquisition of a reduced conditioned response. We noted that the difference did not arise from the reduced response for the first tone-pairing, because the effect was largest at 2 min time point (*a), which was before the first stimulus, and there was no difference in the immediate responses after each foot shock. The effect of the within-subject factor time was similar with each other; therefore, their learning curves during acquisition were also similar. Significant differences in other time points (*b and *c) indicate that SCR (+/−) mice have a reduced learned response. The absence of a significant genotype effect during the complete tone exposure period could be due to the clear absence of differences during the last minute. The possibility that impairment of fear conditioning can be due to anxiety-like behaviors or to an impaired associative mechanism still remains.

### Impairment of contextual fear memory in SCR (+/−) mice

Both at 24 h and 8 day after conditioning, only contextual, but not cued fear learning was impaired in SCR (+/−) mice. The results support a selective effect of *Scrapper* gene deficiency and a specific role of SCRAPPER in the hippocampus, which is consistent with our previous reports [1]. Differences in the context test between 2 genotypes were larger at 24 h after than at 8 days after. The freezing rate at (*f) and (*g) was significant, both with p-value and the freezing rate, are lower than those of data of 24 h after. Of course, the reduction of freezing rate may represent not only the retention but also the extinction effect. Moreover, the freezing rates in context test after 8 days are similar to those in cued test after 8 days, at the first phase pre-conditioned stimulus (tone). It is possible that the absence of SCR (+/−) response is reflection of reduced anxiety.

### SCRAPPER in the hippocampus involved in fear and anxiety

The brain region responsible for fear responses had been researched extensively with selective cytotoxic lesions experiments [37], [41]. As mentioned above, it is widely accepted that the hippocampus and the amygdala play central roles in fear memory. It is reported that the hippocampus plays a role in fear and/or anxiety [42], [43], [44]. Specifically, the lesions of ventral, but not dorsal, hippocampal region affect anxiety; the operated rats had less anxiety than control animal in open field test as well as contextual conditioning, but no spatial learning impairment in the water maze or the elevated T-maze [36], [42]. Those phenotypes are very similar to those of SCR (+/−) shown in this study. Therefore, it is possible that the impairment in SCR (+/−) mice was largely caused by the downregulation of SCRAPPER in ventral hippocampus, Indeed, the impairment of anxiety-related behavior was observed in the open field test [27], [45].

Amygdala is also one of the areas where the anxiety response is processed [31], [32]. We cannot exclude the amygdala involvement in these mice. With post hoc test, there is significance at 4 min time point in cued test (p = 0.0239) and totally SCR (+/−) mice had lower tendency. It is very consistent with the previous report that the most affected brain region in SCR (+/−) mice was hippocampus [1].

### SCRAPPER and the target RIM1 in the learning and memory

We previously reported that RIM1 is the target of the SCRAPPER-dependent UPS pathway [1]. RIM1 is one of the factors modulating synaptic plasticity; it is a well-known protein in the active zone—a specialized presynaptic area for exocytosis— and an essential factor for neurotransmitter release. RIM1 knockout mice show disturbance in the areas of long-term memory and the associative learning deficit in fear conditioning [46], in addition to the alteration of pre-synaptic long-term potentiation (LTP) [17], although RIM1 knockout mice have no consolidation or anxiety-related behavior phenotypes. In the case of RIM S413 or S1548 point mutation mice, neither memory nor anxiety was affected [47]. Because there is no report for RIM1-overexpressed mice, we cannot discuss the effect of upregulated RIM1 protein which is derived from SCRAPPER deficiency.

It is known that fear conditioning links LTP-induced pre- or post-synaptic enhancement of synaptic transmissions in both hippocampus and amygdala [31], [48], [49]. Moreover, RIM1 confers sustained activity and neurotransmitter vesicle anchoring to pre-synaptic Ca^2+^ channels [50], which mediate expression of pre-synaptic LTP [51]. It is possible that SCRAPPER's target involved in fear conditioning is RIM1, although there may be other target synaptic proteins. SCRAPPER-dependent proteasomal degradation can serve in brain circuit building during fear memory formation.

### Fear memory and protein degradation

Our results suggest that the SCRAPPER and possibly SCRAPPER-dependent UPS pathway links to fear memory. At present, little is known about whether protein degradation is involved in the formation of fear memory. Synaptic protein degradation underlies destabilization of retrieved fear memory [52], [53]. Lee *et al.* [52] showed that the postsynaptic proteins were degraded through polyubiquitination after retrieval of contextual fear memory. Kaang *et al.* [53] made the point that the labile state of memory is critical for the reorganization of memory triggered after memory retrieval. They focused on protein degradation in the hippocampus and hippocampus-related memory after memory formation.

In addition to SCRAPPER, kf-1, an E3 ubiquitin ligase, was reported as a suppressor of anxiety [45], [54]. Kf-1 was first identified as a gene with enhanced expression in the cerebral cortex of a sporadic Alzheimer's disease patient [55] and was reported to be involved in the ERAD pathway [56]. Cdh1 is one of the components of the anaphase promoting complex (APC) E3 [57]. Cdh1-heterozygote mice are deficient in contextual fear conditioning, which suggests that Cdh1 is essential to learning and memory [58]. Mice with spontaneous deletion of the ubiquitin C-terminal hydrolase L1 (UCHL1) termed gracile axonal dystrophy (gad) mice showed a reduction in memory in passive avoidance learning and synaptic plasticity [59]. UCHL1 intraperitoneal injections rescue β-amyloid-induced decrease in synaptic function and contextual memory [60]. Analyses of Uchl3 −/− mice suggest that Uchl3 is involved in working memory [61]. In addition, identification of genes expressed in the amygdala showed that ubiquitin related molecules are induced during fear memory formation [62]. The involvement of protein degradation in fear memory formation is now emerging.

### Daily rhythm and protein degradation

Daily rhythms in behavior and physiology are found in almost all organisms. We found SCR (+/−) mice showed hyper activity in the dark period but not in the light period (Fig. 6). This result is interesting because some circadian rhythm regulators are degraded by UPS. For example, it was reported that an F-box protein family FBXL13 binds to and ubiquitinates circadian clock Cryptochrome 1/2 (CRY1/2), as a results, the amount of other clock gene products, period 1/2 (PER1/2), are regulated [63]–[67]. FBXL21 also binds to CRY1, and is highly expressed in the suprachiasmatic nucleus (SCN) [68]. In mammalian brain, the site of the master clock is the hypothalamus: SCN [69] coordinates a network of circadian oscillators that are found throughout the body. Within the brain, components of neural circuits involved in learning and memory, e.g., the hippocampus, exhibit circadian rhythms in gene expression and signaling pathways [70], [71]. Although it is not known whether the circadian rhythm of *Scrapper* mutant mice is disrupted or not because we did not measure it under the complete darkness, the story is attractive that SCRAPPER is involved in the regulation of the daily rhythm of the hippocampus.

### SCR-TG mice did not show significant changes in the behavioral test

The fear conditioning of SCRAPPER-overexpressing TG mice was hardly affected. In addition, SCR-TG mice showed similar results in the general activity test (Table1). The result of reduced tendency in the tail suspension test may be due to the light body mass. Because the exogenous expression of SCR-TG is in hippocampus due to CaMKII promoter [72], [73], we expected that their behavior would change in the learning and memory tasks. However, contrary to our expectations, SCR-TG mice did not show significant differences on tasks including the elevated plus maze test, the Porsolt forced swim test, and the 8-arm radial maze test (Table 1). We suspect that one of the possible reasons is that the exogenous expression level of SCRAPPER was too little to have a crucial effect on the body. The transgenic mice had normal performance in the learning and memory tasks, whereas the RIM1 level was reduced in the hippocampus compared to WT mice [1]. We think that this is because the RIM1 level was reduced but not completely abolished, thus, the phenotypes of SCRAPPER transgenic mice did not mimic those of RIM1 knockout mice.

### Conclusions

In this study, we performed extensive behavioral analysis to determine the involvement of SCRAPPER in these individuals. The present study suggests that the protein level of SCRAPPER and maybe the molecular degradation conferred by SCRAPPER play important roles in contextual fear conditioning, thus hippocampus-dependent fear memory formation.

## Methods

### Animals for behavioral analysis

*Scrapper* knockout mice and transgenic mice were described previously [1]. Male C57/BL6J mice were used for all experiments. Male heterozygous *Scrapper* knockout mice were maintained by backcrossing with C57/BL6J mice. Genetic testing of two SCR-KO mice confirmed that an average of 100.0% of the markers corresponded to C57BL/6J (Genetic Testing Services; Central Institute for Experimental Animals, Kawasaki). We used heterozygous *Scrapper* knockout mice, because it is hard to obtain homozygotes, due to low birthrate and their lethality [1]. Mice were housed four (two pairs of mutant and WT mice) per cage in a room with a 12-hr light/dark cycle with access to food and water ad libitum. Behavioral testing was performed between 9:00 a.m. and 6:00 p.m. After the tests, the apparatus were cleaned with super hypochlorous water to prevent a bias due to olfactory cues. All behavioral tests were conducted in a manner similar to those described previously [26], [74], [75]. All behavioral testing procedures were approved by the Animal Care and Use Committee of Kyoto University Graduate School of Medicine (Permit No., MedKyo 09539).

### Experimental design

Experiments were done in the following sequences; the first group [SCR (+/−) mice]: the general health and neurological screen including wire hang test (GHNS), light/dark transition (LD), open field (OF), elevated plus maze (EP), hot plate (HP), one-chamber social interaction test (SI), Crawley's sociability and preference for social novelty test (CSI), startle response/prepulse inhibition test (PPI), Porsolt forced swim test (PS), gait analysis (GA), rotarod (RR), fear conditioning test (FZ), Barnes maze test (BM), tail suspension test (TS), and social interaction test in home cage (HC-SI); the second group (SCR-TG): GHNS, LD, OF, EP, HP, SI, RR, PPI, PS, FZ, TS and eight-arm radial maze. Each behavioral test was separated from each other at least by 1 day.

### Neurological screen

In the neurological screen, 23 to 28-wk-old male SCR-HKO mice and 20 to 25-wk-old male TG mice were used. The righting, whisker touch, and ear twitch reflexes were evaluated. A number of physical features, including the presence of whiskers or bald hair patches, were also recorded.

Neuromuscular strength was tested with the grip strength test and wire hang test. A grip strength meter (O'Hara & Co., Tokyo, Japan) was used to assess forelimb grip strength. Mice were lifted and held by their tail for grasping a wire grid. The mice were then gently pulled backward by the tail with their posture until they released the grid. Each mouse was tested three times, and the greatest value measured was used for statistical analysis. In the wire hang test, the mouse was placed on a wire mesh that was then inverted and waved gently, so that the mouse gripped the wire. Latency to fall was recorded, with a 60 s cut-off time.

### Rotarod test

Rotarod test was performed with 30 to 35-wk-old male SCR-HKO mice and 22 to 27-wk-old male TG mice. Motor coordination and balance were tested with the rotarod test. The rotarod test, using an accelerating rotarod (UGO Basile Accelerating Rotarod), was performed by placing mice on rotating drums (3 cm diameter) and measuring the time each animal was able to maintain its balance on the rod. The speed of the rotarod accelerated from 4 to 40 rpm over a 5-min period.

### Open field test

Open field test was performed with 23 to 28-wk-old male SCR-HKO mice and 21 to 26-wk-old male TG mice. Locomotor activity was measured using an open field test. Each mouse was placed in the center of the open field apparatus (40×40×30 cm; Accuscan Instruments, Columbus, OH). Total distance traveled (in cm), vertical activity (rearing measured by counting the number of photobeam interruptions), time spent in the center, the beam-break counts for stereotyped behaviors, and number of fecal boli were recorded. Data were collected for 120 min.

### Light/dark transition test

Light/dark transition test was performed with 23 to 28-wk-old male SCR-HKO mice and 21 to 25-wk-old male TG mice. The apparatus used for the light/dark transition test consisted of a cage (21×42×25 cm) divided into two sections of equal size by a partition containing a door (O'Hara & Co., Tokyo, Japan). One chamber was brightly illuminated (390 lux), and the other chamber was dark (2 lux). Mice were placed into the dark side at first, and then allowed to move freely between the two chambers with the door open for 10 min. The total number of transitions between chambers, time spent in each side, first latency to enter the light side and distance traveled were recorded automatically.

### Social interaction test in a novel environment

Social interaction test was performed with 24 to 29-wk-old male SCR-HKO mice and 22 to 27-wk-old male TG mice. For social interaction test in a novel environment two mice of identical genotypes that were previously housed in different cages, were placed into a box together (40×40×30 cm) and allowed to explore freely for 10 min. Social behavior was monitored by a CCD camera. Analysis was performed automatically using Image SI software. Total duration of contact, the number of contacts, the number of active contacts, mean duration per contact, and total distance traveled were measured. The number of active contacts was defined as following procedure. Images were captured at one frame per second, and distance traveled between two successive frames was calculated for each mouse. If the two mice contacted each other and the distance traveled by either mouse was longer than 5 cm, the behavior was considered as ‘active contact’.

### Social interaction test in a novel environment (one-chamber social interaction test)

In the social interaction test, two mice of identical genotypes that were previously housed in different cages were placed in a box together (40×40×30 cm) and allowed to explore freely for 10 min. Social behavior was monitored with a CCD camera. Analysis was performed automatically using Image SI software (see below ‘Data analysis’). The total number of contacts, total duration of active contacts, total contact duration, mean duration per contact, and total distance traveled were measured. The active contact was defined as follows. Images were captured at 1 frame per second, and distance traveled between two successive frames was calculated for each mouse. If the two mice contacted each other and the distance traveled by either mouse was longer than 2 cm, the behavior was considered as ‘active contact’.

### Social interaction test in home cage

Social interaction monitoring in the home cage was conducted as previously described [22]. The system comprised the home cage (29×18×12 cm) and a filtered cage top, separated by a 13-cm-high metal stand containing an infrared video camera attached at the top of the stand. Two mice of the same genotype that had been housed separately were placed together in a home cage. Their social behavior was then monitored for 1 week. Images from each cage were captured at a rate of one frame per second. Social interaction was measured by counting the number of particles detected in each frame: two particles indicated that the mice were not in contact with each other; and one particle (i.e., the tracking software could not distinguish two separate bodies) indicated contact between the two mice. We also measured locomotor activity during these experiments by quantifying the number of pixels that changed between each pair of successive frames. Analysis was performed automatically using Image HA software (see ‘Data analysis’).

### Crawley's sociability and preference for social novelty test

The test for sociability and preference for social novelty was conducted as previously described [27] with 26 to 31-wk-old male SCR-HKO mice. The apparatus comprised a rectangular, three-chambered box and a lid containing an infrared video camera (Ohara & Co.). Each chamber was 20×40×22 cm and the dividing walls were made from clear Plexiglas, with small square openings (5×3 cm) allowing access into each chamber. An unfamiliar C57BL/6J male (stranger 1) that had no prior contact with the subject mouse was placed in one of the side chambers. The placement of stranger 1 in the left or right side chambers was systematically alternated between trials. The stranger mouse was enclosed in a small, circular wire cage that allowed nose contact between the bars, but prevented fighting. The cage was 11 cm high, with a bottom diameter of 9 cm and bars spaced 0.5 cm apart. The subject mouse was first placed in the middle chamber and allowed to explore the entire social test box for 10-min. The amount of time spent within a 5-cm distance of the wire cage and in each chamber. At the end of the first 10 min, each mouse was tested in a second 10-min session to determine quantity of social preference for a new stranger. A second, unfamiliar mouse was placed in the chamber that had been empty during the first 10-min session. This second stranger was enclosed in an identical small wire cage. The test mouse had a choice between the first, already-investigated unfamiliar mouse (stranger 1), and the novel unfamiliar mouse (stranger 2). As described above, the amount of time spent within a 5-cm distance of each wire cage and in each chamber during the second 10-min session was recorded. The stranger mice used in this experiment were 8 to 12-wk-old C57BL/6J male mice, not littermates. Analysis was performed automatically using Image CSI software.

### Contextual and cued fear conditioning

Fear conditioning test was performed with 32 to 38-wk-old male SCR-HKO mice and 25 to 30-wk-old male TG mice. On the training day, each mouse was placed into a conditioning chamber (10.5×10.5×10.5 cm; O'Hara & Co., Tokyo, Japan) and allowed to explore freely for 2 min. A tone (75 dB) was presented as the conditioned stimulus for 30 s followed by a 2 s mild foot shock (0.35 mA) as the unconditioned stimulus. One or two more tone-shock pairs were given at 2 min intervals and the animal was returned to its home cage 30 s after the last pair. At 24 h after the conditioning session, the mice were placed back into the conditioning chamber for 5 min and their freezing behavior was measured in context. At 1 h after context testing, the mice were placed into a different, white Plexiglas chamber for 3 min and then the tone was turned on for 3 min. Freezing during the first and subsequent 3 min intervals was recorded. At 8 days after conditioning, the freezing behavior was measured in the same manner as recording at 24 h after.

### Elevated plus-maze test

Elevated plus-maze test was performed with 24 to 29-wk-old male SCR-HKO mice and 21 to 26-wk-old male TG mice. The elevated plus-maze (O'Hara & Co., Tokyo, Japan) consisted of two open arms (25×5 cm) and two enclosed arms of the same size, with 15-cm high transparent walls. The arms and central square were made of white plastic plates and were elevated to a height of 55 cm above the floor. To minimize the likelihood of animals falling from the apparatus, 3-mm high plastic ledges were provided for the open arms. Arms of the same type were arranged at opposite sides to each other. Each mouse was placed in the central square of the maze (5×5 cm), facing one of the closed arms. Mouse behavior was recorded during a 10-min test period. The number of entries into, and the time spent in open and enclosed arms, were recorded. For data analysis, we used the following four measures: the percentage of entries into the open arms, the time spent in the open arms (s), the number of total entries, and total distance traveled (cm). Data acquisition and analysis were performed automatically using Image EP software.

### Hot plate test

Hot plate test was performed with 24 to 29-wk-old male SCR-HKO mice and 22 to 27-wk-old male TG mice. The hot plate test was used to evaluate sensitivity to a painful stimulus. 11-wk-old mice were placed on a 55.0 (±0.3) °C hot plate (Columbus Instruments), and latency to the first hind-paw response was recorded. The hind-paw response was defined as either a foot shake or a paw lick.

### Startle response/prepulse inhibition tests

Startle response/prepulse inhibition test was performed with 26 to 31-wk-old male SCR-HKO mice and 24 to 28-wk-old male TG mice. A startle reflex measurement system was used (O'Hara & Co., Tokyo, Japan) to measure startle response and prepulse inhibition. A test session began by placing a mouse in a plastic cylinder where it was left undisturbed for 10 min. White noise (40 ms) was used as the startle stimulus for all trial types. The startle response was recorded for 140 ms (measuring the response every 1 ms) starting with the onset of the prepulse stimulus. The background noise level in each chamber was 70 dB. The peak startle amplitude recorded during the 140 ms sampling window was used as the dependent variable. A test session consisted of six trial types (i.e., two types for startle stimulus only trials, and four types for prepulse inhibition trials). The intensity of the startle stimulus was 110 or 120 dB. The prepulse sound was presented 100 ms before the startle stimulus, and its intensity was 74 or 78 dB. Four combinations of prepulse and startle stimuli were used (74–110, 78–110, 74–120, and 78–120). Six blocks of the six trial types were presented in pseudorandom order such that each trial type was presented once within a block. The average inter-trial interval was 15 s (range: 10–20 s).

### Porsolt forced swim test

Porsolt forced swim test was performed with 27 to 32-wk-old male SCR-HKO mice and 24 to 29-wk-old male TG mice. The apparatus consisted of four plastic cylinders (20 cm height_10 cm diameter). The cylinders were filled with water (23°C) up to a height of 7.5 cm. Mice were placed into the cylinders, and their behavior recorded over a 10-min test period. Data acquisition and analysis were performed automatically, using Image PS software (see ‘Image Analysis’). Distance traveled was measured by Image OF software (see ‘Image Analysis’) using stored image files.

### Eight-arm radial maze test

Eight-arm radial maze test was performed with 23 to 28-wk-old male SCR-HKO mice and 21 to 26-wk-old male TG mice. Fully-automated eight-arm radial maze apparatuses (O'Hara & Co., Tokyo, Japan) were used. The floor of the maze was made of white plastic, and the wall (25 cm high) consisted of transparent plastic. Each arm (9×40 cm) radiated from an octagonal central starting platform (perimeter 12×8 cm) like the spokes of a wheel. Identical food wells (1.4 cm deep and 1.4 cm in diameter) with pellet sensors were placed at the distal end of each arm. The pellets sensors were able to automatically record pellet intake by the mice. The maze was elevated 75 cm above the floor and placed in a dimly-lit room with several extra-maze cues. During the experiment, the maze was maintained in a constant orientation. One week before pretraining, animals were deprived of food until their body weight was reduced to 80% to 85% of the initial level. Pretraining started on the 8th day. Each mouse was placed in the central starting platform and allowed to explore and consume food pellets scattered on the whole maze for a 30-min period (one session per mouse). After completion of the initial pretraining, mice received another pretraining to take a food pellet from each food well after being placed at the distal end of each arm. A trial was finished after the mouse consumed the pellet. This was repeated eight times, using eight different arms, for each mouse. After these pretraining trials, actual maze acquisition trials were performed. In the spatial working memory task of the eight-arm radial maze, all eight arms were baited with food pellets. Mice were placed on the central platform and allowed to obtain all eight pellets within 25 min. A trial was terminated immediately after all eight pellets were consumed or 25 min had elapsed. An ‘arm visit’ was defined as traveling more than 5 cm from the central platform. The mice were confined at the center platform for 5 s after each arm choice. The animals went through one trial per day. For each trial, arm choice, latency to obtain all pellets, distance traveled, number of different arms chosen within the first eight choices, the number of arm revisited, and omission errors were automatically recorded. In the reference memory task of the eight-arm radial maze, one of the eight arms was consistently baited with one food pellet in the food well and a trial was terminated immediately after the one pellet was consumed. Data acquisition, control of guillotine doors, and data analysis were performed by Image RM software (see ‘Image analysis’).

### Locomotor activity monitoring in home cage

The system that automatically analyzes the locomotor activity of mice in their home cage was used [76]. The system contains a home cage (29×18×12 cm) and a filtered cage top, separated by a 13-cm-high metal stand containing an infrared video camera, which is attached to the top of the stand. Each mouse was individually housed in each home cage, and monitored. Images from each cage were captured at a rate of one frame per second, and distance traveled was measured automatically using Image HA software (see ‘Image analysis’).

### Gait analysis (front and hind paws)

Gate analysis test was performed with 27 to 32-wk-old male SCR-HKO mice. The gait of the adult mouse during spontaneous walk/trot locomotion at velocities has been analyzed using simultaneous video and reaction force analysis. Equivalent stride times for fore and hind paws were composed of a shorter stance and a longer swing time. Peak vertical reaction force increases with decreasing stance time, with that for the forelimb being about 5% greater than that for the hind paws across the whole stance time range studied.

### Barnes maze

Barnes maze test was performed with 34 to 52-wk-old male SCR-HKO mice. The Barnes maze consisted of a white, acrylic, circular disk 90 cm in diameter with 12 equally spaced holes (5-cm diameter) located 5 cm from the edge, as previously described [77]. Each of the holes could be opened or closed by means of a sliding, white acrylic door that fit snugly under the hole. A black acrylic escape box (8×8×8 cm), to which the mice could gain access by way of a white acrylic ramp, could be fitted under any of the holes in place of the door. From the center of the maze, the white acrylic ramp looked identical to the white acrylic sliding doors used to block the other 11 holes. Thus the mice could not visually discriminate the escape hole location from the other holes from most points on the maze. However, when mice were situated adjacent to the escape hole, they could discriminate the escape from nonescape locations either tactilely or visually by looking down the tunnel into the black escape box. The maze was raised 56 cm from the floor and rested on a pedestal that enabled it to be rotated 360° on a horizontal plane. The black acrylic start box was a 13×13×13 cm bottomless cube with a hinged lid and a handle for easy lifting. Trials were recorded by using a CCD camera and were analyzed by using the public domain NIH Image program (rsb.info.nih.gov/nih-image), using a macro written specifically for the Barnes maze [27]. This software allows automated tracking and analysis of escape paths. The target zone was defined as the escape hole and 1 cm around it.

### Tail suspension test

Tail suspension test was performed as described previously [78] with 51 to 56-wk-old male SCR-HKO mice and 35 to 41-wk-old male TG mice for a 10-min test session. Mice were suspended 30 cm above the floor in a visually isolated area by adhesive tape placed ∼1 cm from the tip of the tail, and their behavior was recorded over a 10-min test period. Data acquisition and analysis were performed automatically, using Image TS software.

### Image analysis

The applications used for the behavioral studies (Image LD, Image EP, Image RM, Image FZ, Image SI, Image TS and Image HA) were based on the public domain NIH Image program (developed at the U.S. National Institutes of Health and available on the Internet at http://rsb.info.nih.gov/nih-image/) and ImageJ program (http://rsb.info.nih.gov/ij/), which were modified for each test by Tsuyoshi Miyakawa (available through O'Hara & Co., Tokyo, Japan).

### Statistical analysis

Statistical analysis was conducted using StatView (SAS Institute, Cary, NC). Data were analyzed by two-way ANOVA, or two-way repeated measures ANOVA, unless noted otherwise. Values in tables and graphs were expressed as mean ± s.e.m. Post-hoc comparisons were performed using Fisher's Protected Least Significant Difference (Fisher's PLSD) multiple comparisons. Genotype × Time was calculated by a repeated measures ANOVA.

## Supporting Information

Table S1 Comparison between *Scrapper*-knockout [SCR (+/−)] - and wild-type (WT) mice or *Scrapper*-transgenic (SCR-TG) - and WT mice. The p values indicate genotype effect in two-way ANOVA. ANOVA F values are given for the comparison of phenotypes between 2 genotypes.
(XLS)

Table S2 Crawley's sociability and preference for social novelty test between *Scrapper*-knockout [SCR (+/−)] - and wild-type (WT) mice. The p values indicate genotype effect in two-way ANOVA.
(XLS)
