# Correction: Synaptic E3 Ligase SCRAPPER in Contextual Fear Conditioning: Extensive Behavioral Phenotyping of *Scrapper* Heterozygote and Overexpressing Mutant Mice

**DOI:** 10.1371/annotation/1bf38b37-ee2e-41fb-bad4-ed719b07c6c0

**Published:** 2011-06-07

**Authors:** Ikuko Yao, Keizo Takao, Tsuyoshi Miyakawa, Seiji Ito, Mitsutoshi Setou

The correct funding information is: "This study is supported by: Research Grants for PRESTO and SENTAN from JST, Grant-in-Aid for Scientific Research on Priority Areas Molecular Brain Science and Research on Pathomechanisms on Brain Disorders from MEXT. The funders had no role in study design, data collection and analysis, decision to publish, or preparation of the manuscript."

